# Evaluating Multi-Temporal Sentinel-1 and Sentinel-2 Imagery for Crop Classification: A Case Study in a Paddy Rice Growing Region of China

**DOI:** 10.3390/s26020586

**Published:** 2026-01-15

**Authors:** Rui Wang, Le Xia, Tonglu Jia, Qinxin Zhao, Qiuhua He, Qinghua Xie, Haiqiang Fu

**Affiliations:** 1 School of Geography and Information Engineering, China University of Geosciences (Wuhan), Wuhan 430074, China; 1202410866@cug.edu.cn (R.W.); jtlcug@cug.edu.cn (T.J.); qinxinzhao@cug.edu.cn (Q.Z.); xieqh@cug.edu.cn (Q.X.); 2 Technology Innovation Center for Ecological Conservation and Restoration in Dongting Lake Basin, Ministry of Natural Resources, Changsha 410083, China; hqhhnrs@hotmail.com; 3 School of Geosciences and Info-Physics, Central South University, Changsha 410083, China; haiqiangfu@csu.edu.cn

**Keywords:** Sentinel-1, Sentinel-2, polarimetric SAR decomposition, crop classification, paddy rice

## Abstract

**Highlights:**

**What are the main findings?**

- The decomposition parameters $m_{v}$ derived from the dual-polarization model-based decomposition can effectively discriminate different crop types.
- Multi-temporal optical data with low cloud cover can effectively support crop classification. Incorporating dual-polarimetric SAR data further enhances the classification accuracy, particularly for rice and corn.

**What is the implication of the main finding?**

- A practical classification strategy was proposed for crop type identification.
- A 10-m-resolution thematic map was produced to classify crops in the study area.

**Abstract:**

Information on crop planting structure serves as a key reference for crop growth monitoring and agricultural structural adjustment. Mapping the spatial distribution of crops through feature-based classification serves as a fundamental component of sustainable agricultural development. However, current crop classification methods often face challenges such as the discontinuity of optical data due to cloud cover and the limited discriminative capability of traditional SAR backscatter intensity for spectrally similar crops. In this case study, we assess multi-temporal Sentinel-1 and Sentinel-2 Satellite images for crop classification in a paddy rice growing region in Helonghu Town, located in the central region of Xiangyin County, Yueyang City, Hunan Province, China (28.5° N–29.0° N, 112.8° E–113.2° E). We systematically investigate three key aspects: (1) the classification performance using optical time-series Sentinel-2 imagery; (2) the time-series classification performance utilizing polarimetric SAR decomposition features from Sentinel-1 dual-polarimetric SAR images; and (3) the classification performance based on a combination of Sentinel-1 and Sentinel-2 images. Optimal classification results, with the highest overall accuracy and Kappa coefficient, are achieved through the combination of Sentinel-1 (SAR) and Sentinel-2 (optical) data. This case study evaluates the time-series classification performance of Sentinel-1 and Sentinel-2 data to determine the optimal approach for crop classification in Helonghu Town.

## 1. Introduction

Accurate and timely information on crop distribution is essential for agricultural production management, yield estimation, and sustainable land use planning [1,2]. Crop classification is essential for agricultural monitoring, providing the foundation for tracking crop growth, optimizing planting structures, and ensuring food security, while also supporting agricultural research, resource management, breeding innovation, and sustainable development [3,4]. The development of advanced crop distribution mapping algorithms is critical for establishing a robust and generalizable model applicable to large-scale agricultural monitoring via remote sensing [5,6]. Given the increasing pressures of population growth, global environmental change, and limited arable land resources, obtaining reliable and efficient crop classification has emerged as an urgent applied research challenge [7,8]. Crop growth dynamics are governed by critical factors including soil conditions, irrigation systems, and climatic variables [9]. Precise crop classification—essential for growth optimization due to species-specific environmental adaptability—forms the foundation for effective yield estimation and prediction [10,11,12].

Traditional crop classification approaches often rely exclusively on intensive ground surveys to characterize farmland attributes, posing significant economic and temporal constraints. As a non-contact information acquisition tool, remote sensing is extensively applied in agriculture and land management [10]. Remote sensing data with spatiotemporal heterogeneity substantially enhance the classification accuracy of land cover types, thereby enabling more precise mapping and analysis of farmland distribution [13]. Compared to conventional methods, remote sensing offers distinct advantages: (1) simultaneous large-scale observation with enhanced analytical timeliness; (2) reduced susceptibility to weather and environmental interference, lowering operational costs while improving reliability; and (3) establishment of comprehensive farmland production databases.

Optical remote sensing for crop classification has matured over decades. For land cover and land use classification, multi-temporal high-resolution optical imagery has become a critical tool—this shift has been enabled by the expanding repository of remote sensing satellite data. The calculation of the Normalized Difference Vegetation Index (NDVI) serves as a reliable method for extracting critical information on surface vegetation [14,15], and this index is widely used to predict key growth stages, such as the grain-filling and ripening stages of rice, providing robust technical support for the development of crop growth models. Rice spectral response varies across phenological stages. During early emergence and tillering, sparse canopy and low biomass result in low red and NIR reflectance, yielding lower NDVI and EVI values. As the canopy thickens and chlorophyll increases during tillering and jointing, NIR reflectance and VIs peak [16,17]. In the heading and flowering stages, with full canopy closure and maximum LAI, NIR reflectance and VIs reach their highest values, strongly correlating with yield [18,19,20]. In the grain filling stage, chlorophyll decreases and the canopy yellows, causing a drop in NIR and an increase in red reflectance, leading to a decline in NDVI and EVI. At maturity, as grains ripen and leaves yellow, red and SWIR reflectance increase, while NIR decreases, with a distinct red-edge shift [21]. These spectral changes are critical for differentiating rice growth stages and are widely used in optical remote sensing phenology studies. Moreover, the integration of NDVI and the Normalized Water Index (NDWI) has demonstrated promising results in crop recognition, particularly for rice [22]. While optical remote sensing technology exhibits strengths such as fine resolution and well-defined feature attributes, its effectiveness is often limited by weather conditions. For instance, crops are predominantly cultivated in subtropical regions, where the planting season often overlaps with the rainy season, posing significant challenges for monitoring. The persistent influence of environmental factors may render the accumulation of temporal imagery insufficient to significantly enhance crop classification accuracy.

Synthetic Aperture Radar (SAR) penetrates clouds, fog, and snow, enabling direct observation of surface targets [23]. This all-weather capability drives its increasing adoption in crop classification studies [24,25,26,27]. In addition, beyond these conventional applications, advanced polarimetric decomposition techniques have significantly enhanced SAR’s sensitivity to vegetation structure [28,29]. Researchers have discovered that polarimetric SAR features exhibit higher sensitivity to crop structure and biophysical characteristics than optical vegetation index features [19]. The full polarimetric SAR signals show a strong potential in the application of effective differentiation of various crops types [30]. In agriculture, model-based polarimetric decomposition based on physical scattering models quantifies parameters like volume scattering power and surface scattering power, providing a mechanistic basis for crop classification. Here, volume scattering is correlated with crop biomass and height characterizes canopy multiple scattering [31], while surface scattering reflects soil interface scattering [32]. Studies on scattering mechanisms based on the two-dimensional H-α eigenspace have shown that significant progress has been made in the differentiation of target types using the eigenvalues of the coherence matrix and its associated eigenvectors [33,34,35]. The H/A/α/ decomposition parameters constitute a robust analytical framework for polarimetric SAR image interpretation and has been extensively applied across diverse areas, including crop classification, land use classification [34,36]. These parameters outperform traditional backscatter coefficients by isolating scattering mechanisms, thereby improving classification accuracy. Current Sentinel-1-based crop classification methodologies predominantly rely on backscatter coefficients and H/A/α/ decomposition features [37,38,39,40], underutilizing advanced polarimetric information derived from model-based decomposition approaches.

The contribution of single SAR images to rice mapping needs to be improved [41], and future research needs to deepen the coupling of scattering mechanisms and agronomic parameters to promote the paradigm shift in crop classification from two-dimensional mapping to three-dimensional synergistic perception of structure and physiological state. Long-time-series SAR data can effectively monitor the scattering characteristic changes during crop growth [42,43], so it is advantageous to utilize long-time-series images for crop classification. Current Sentinel-1-based crop classification methodologies predominantly rely on backscatter coefficients and H/A/α/ decomposition features [37,38,39,40], underutilizing advanced polarimetric information derived from model-based decomposition approaches. Therefore, fully utilizing multi-temporal polarization decomposition features can effectively characterize crop structural changes and further enhance classification performance [44,45].

The main objectives of this case study are summarized as follows: (1) To assess the accuracy, temporal regularity, and classification effectiveness of multi-temporal Sentinel-2 imagery for agricultural crop classification with different crops. (2) To evaluate the classification performance, temporal regularity, and crop-recognition capabilities of multi-temporal Sentinel-1 data, emphasizing the contribution of model-based polarimetric decomposition features. (3) From a practical perspective, to determine the most suitable crop classification strategy for the research region by comparatively analyzing the Sentinel-1 and Sentinel-2 images.

## 2. Materials and Methods

### 2.1. Study Area

**Figure 1:**
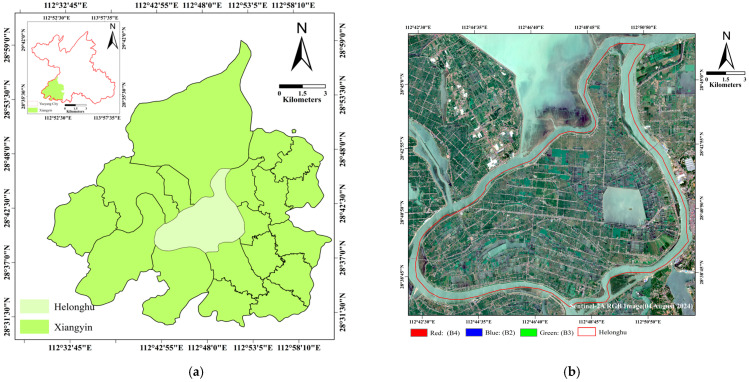
(**a**) Overview of the research area; (**b**) A Sentinel-2B true-color (RGB) composite image (acquisition date 4 August 2024). (Red: B4, Green: B3, Blue: B2).

The town of Helonghu is located in the center of Xiangyin County, Yueyang City, Hunan Province (28.5° N–29.0° N, 112.8° E–113.2° E), as shown in Figure 1, which belongs to a typical lake-wetland ecosystem. The town holds significant geographic and economic importance in the region due to its abundant water resources and unique ecological environment. These favorable ecological conditions and soil quality provide an optimal environment for the cultivation of various crops, including rice, soybeans, corn, and ramie. Rice exhibits a remarkably consistent cultivation timeline: sowing (early–mid-May), growth (mid-May–August), and harvesting (August–late September). Corn and soybeans display a shorter cycle, with sowing in early April and harvest by late August. Ramie (Boehmeria nivea), a perennial herbaceous plant highly valued for its high-quality fiber, is also an important economic crop in Helonghu Town. Its planting and harvesting seasons span from mid-April to early September.

### 2.2. Sentinel Data

For SAR data in this experimental task, images acquired by Sentinel-1A (a satellite with a 12-day revisit cycle) were employed. Comprehensive SAR satellite metadata is detailed in Table 1. As a member of the European Space Agency (ESA)’s Sentinel program, this satellite was designed to supply high-quality data for global environmental monitoring. It is widely applied in natural disaster monitoring, resource management, and urban planning. The satellite is characterized by its extensive coverage and substantial data volume [46,47]. It operates by transmitting C-band signals to the ground, enabling radar image data acquisition for Earth observation under any temporal and weather conditions. The Sentinel-1 data format used in this experiment was Single-Look Complex (SLC), with basic information shown in Table 1. The temporal range of the collected imagery extended over approximately seven months (from 28 February through 19 September 2024), yielding 12 images for analysis. For this experimental task, the Sentinel-1 data utilized the Interferometric Wide Swath (IW) mode as its imaging configuration. This mode is primarily employed for terrestrial observations and satisfies the accuracy requirements of this experiment. In single-look mode, the spatial resolution of the IW mode for surface observation can reach 5 m × 20 m, and its imaging swath width can extend up to 250 km per strip. Employing the progressive scan-based Terrain Observation SAR (TOPSAR) approach, the IW mode can effectively acquire three sub—areas of the study region in terrain variation observation, which is unattainable using traditional scanning radar techniques. Moreover, the spatial resolution of SAR imaging and data processing efficiency are enhanced by improvements in the radar scanning mode and data processing algorithms within the TOPSAR technique.

The study incorporated multispectral imagery sourced from the Sentinel-2A and Sentinel-2B missions. Operating in a coordinated orbital pattern, this satellite constellation facilitates comprehensive planetary monitoring through an optimized revisit cycle. The spatial resolution of the images varies across different bands, with the multispectral band offering a wealth of spectral information. Notably, the B2,B3,B4,B8 bands achieve a spatial resolution of 10 m. Each image has less than 20% cloud cover. Detailed information on the Sentinel-2 satellite bands is presented in Table 2. A series of 8 qualified Sentinel-2 images formed the basis of the temporal analysis, spanning the period from 12 January 2024, to 18 September 2024.

All Sentinel-1 and Sentinel-2 satellite imagery used in this study was obtained from the official European Space Agency (ESA) Copernicus Open Access Hub. For Sentinel-2 data, only Level-2A products with less than 20% cloud cover were selected to ensure the quality of the optical images and minimize the influence of atmospheric conditions on classification accuracy.

### 2.3. Field Data Collection

**Figure 2:**
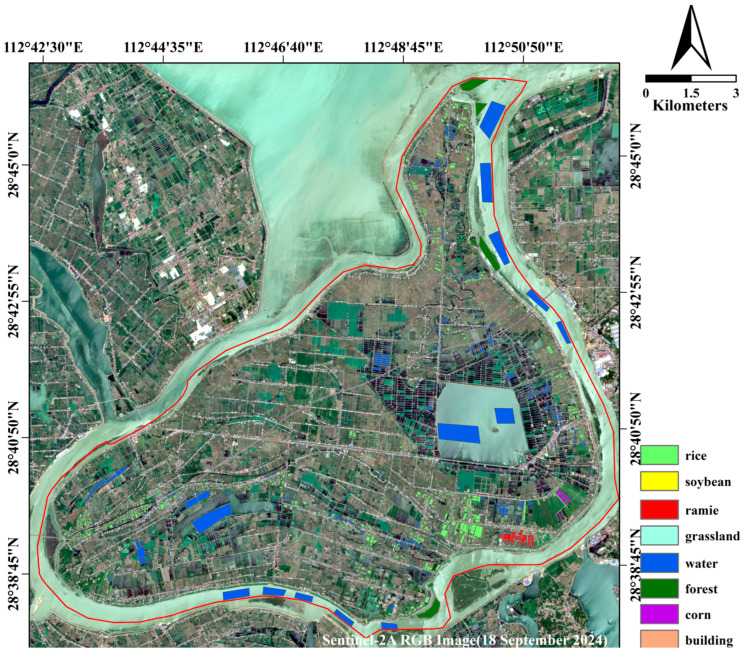
Map of Field Collection Data in Helonghu Township.

The dataset encompassed crop types and location, which provided a substantial amount of ground truth for the subsequent crop classification. A total of 8 types of data were collected, including 4 types of crops: rice, corn, soybean, and ramie. Specifically, 1227 rice polygons, 19 corn polygons, 251 soybean polygons, and 67 ramie polygons were gathered. The distribution and information of the measured data are depicted in Figure 2 and detailed in Table 3, respectively.

In the study area, rice cultivation is divided into double-cropping rice and single cropping rice. Double-cropping rice is further subdivided into double-cropping early rice and double-cropping late rice. In general, the double-cropping early rice has a preparatory sowing period from 15 to 20 March, followed by sowing from 20 to 25 March. The seedling stage lasts for approximately 25–30 days. Transplanting takes place from 20 to 25 April, while the tillering stage occurs from 1 to 12 May. The jointing and heading stages typically occur from mid-May to mid-June, with the grain-filling period lasting about one month. Consequently, early double-cropping rice usually reaches maturity by mid-July.

The double-cropping late rice is sown and prepared for planting in mid-June, with transplanting carried out in mid-July. The tillering stage occurs from late July to early August, followed by the panicle initiation stage. Panicle elongation lasts for approximately one month, and the grain-filling period extends from late September to early October, lasting 40–45 days. Therefore, double-cropping late rice typically reaches maturity by around October 20. Single-cropping rice is prepared for plowing before 10 May, with sowing taking place in mid-to-late May. The seedling stage lasts for approximately 25–30 days. Transplanting is carried out in early to mid-June, followed by the tillering stage in mid-to-late June. The jointing and panicle elongation stage begins in mid-July, and the grain-filling stage lasts for about 40–45 days. The crop reaches maturity by the end of September.

**Figure 3:**
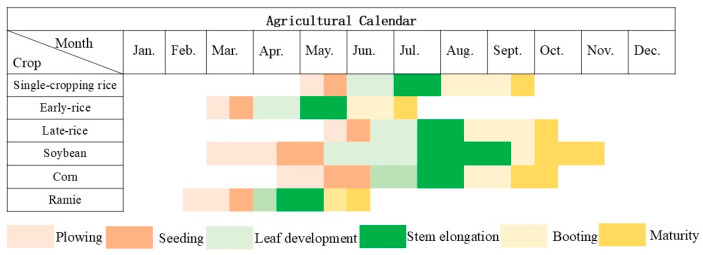
Agricultural Calendar of Rice, Soybean, Corn, and Ramie.

Field surveys were conducted from 21 to 23 July 2024, corresponding to the mid-to-late growing season of the main crops in the study area. During this period, the double-cropping early rice had already been harvested. Due to variations in actual planting conditions, the double-cropped late rice remained in the vegetative growth stage, with the plants appearing relatively sparse, whereas the single-cropped rice had entered the jointing and panicle elongation stage, exhibiting dense growth. Soybeans and corn were in their reproductive growth stages, with soybeans approaching the pod filling stage and corn nearing tasseling and silking [48]. As a perennial fiber crop, ramie had already entered the late growth stage, just prior to harvest [49]. These phenological conditions formed distinct spectral and structural characteristics, which are crucial for the interpretation of remote sensing classification results. The growth stages corresponding to different crops are illustrated in Figure 3.

### 2.4. Random Forest Classification Algorithm

In this experiment, we employed the Random Forest algorithm, a prevalent ensemble learning technique commonly used for classification and regression tasks [50]. By developing an ensemble of decision trees and synthesizing their predictions, this methodology strengthens both the precision and reliability of the model, thereby reducing overfitting and improving generalization performance. Random Forest was selected as the classifier for all experiments to minimize algorithmic variance. This allows the study to focus specifically on evaluating the contribution of different feature sets, particularly the polarimetric decomposition parameters, to the classification accuracy. The Random Forest incorporated 150 decision trees. The reference dataset was divided into training and testing sets using a stratified random sampling strategy based on the total pixel count of each class, with a ratio of 7:3. This ensured that the sample distribution in both subsets was representative of the overall dataset variability.

### 2.5. Data Preprocessing

#### 2.5.1. Data Processing Flow

We used the official ESA professional software SNAP 9.0.0 for the pre-processing of the Sentinel data [51]. For the Sentinel-1 data, it mainly involve Image split, Calibrate, Deburst, Extraction of $C_{2}$ matrix, Multi-looking and Speckle filter (9 × 9 boxcar) [52], and finally Geocoding resampling to 10 m resolution. For the Sentinel-2 data, we selected Level 2A imagery, which had already undergone atmospheric correction, so it is only necessary to resample the eight time-series images of the 12 bands to a 10 m spatial resolution, consistent with Bands 2, 3, 4, and 8 [53]. This resampling process ensured that the spatial resolution was consistent across all images, facilitating subsequent analysis.

**Figure 4:**
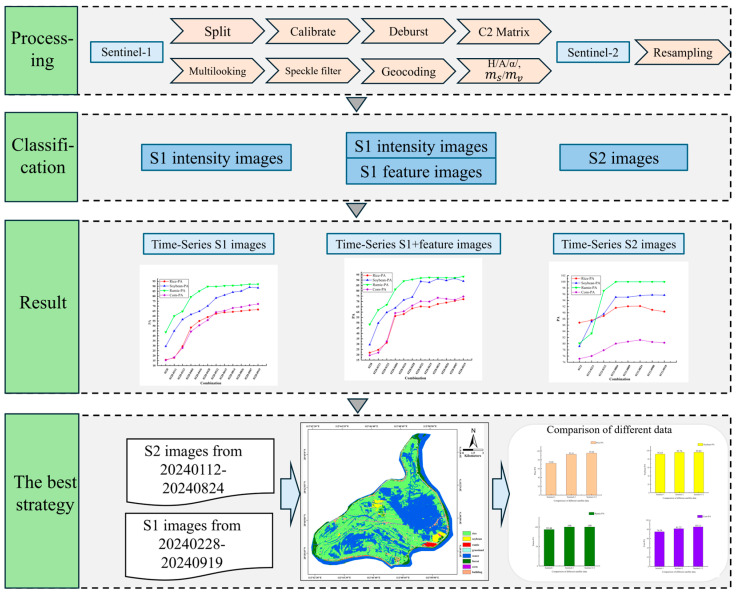
General technical flowchart of this study.

Image fusion [13,24] is a powerful technique that combines data from different sources to enhance the feature and spectral information. In this experiment, the optical dataset consisted of 96 bands after construction, while the SAR dataset comprised 84 bands. By adding H/A/α/ and the model-based dual-polarization decomposition features, and merging these two datasets, we construct a comprehensive feature dataset containing 180 bands. The rich spectral information and prominent feature characteristics of this dataset significantly improved the subsequent classification accuracy. The overall experimental workflow is illustrated in Figure 4.

#### 2.5.2. Polarimetric Decomposition Features

This experiment derived five distinct polarimetric decomposition parameters. The $C_{2}$ matrix, which contains information about radar signal returns at different time points or under varying conditions, serves as a crucial basis for extracting surface features and analyzing changes [54,55]. It is calculated as follows (Equation (1)):

(1) $$C_{2\;}=\;\left[\begin{array}{cc} \langle {\left|S_{VV}\right|}^{2}\rangle & \langle S_{VV}S_{VH}^{*}\rangle \\ \langle S_{VH}S_{VV}^{*}\rangle & \langle {\left|S_{VH}\right|}^{2}\rangle \end{array}\right]$$

The main diagonal elements (“$C_{11}$” and “$C_{22}$”) of the covariance matrix “$C_{2}$” represent the *VV* and *VH* polarimetric scattering signals, respectively.

(1) Cloude-Pottier decomposition

The Cloude-Pottier decomposition (also named H/A)/α) is an eigenvalue–eigenvector-based method [56], which can extract parameters from the polarimetric coherence matrix (Equation (2)) to quantify scattering randomness and dominant mechanisms.

(2) $$\begin{array}{c} T_{2\;}=U_{2}\Sigma U_{2}^{-1}=\sum_{i=1}^{2}\lambda_{i}T_{2i}=\sum_{i=1}^{2}\lambda_{i}u_{i}u_{i}^{*T} \end{array}$$

This decomposition method has three decomposition parameters, the entropy of polarization (H), degree of anisotropy (A), and mean scattering angle (α), with the expressions (Equations (3)–(5)):

(3) $$H\;=\;\sum_{i\;=\;1}^{n}-\;P_{i}{log}_{n}P_{i},\;0\leq H\leq 1$$

(4) $$A=\frac{\lambda_{2}-\lambda_{3}}{\lambda_{2\;}+\lambda_{3}}$$

(5) $$\alpha =\sum_{i=1}^{n}P_{i}\alpha_{i}$$

(2) Dual-polarization model-based decomposition

Mascolo et al. [57] established a decomposition methodology specifically designed for dual-polarization SAR data, utilizing model-based scattering mechanisms. All current polarization decomposition models consist of a body scattering model and a method to extract one or more (polarization) residual terms, which is not possible due to the limited information on the dual polarization for this complete decomposition. Therefore, they simulated the Stokes vector by combining the body scattering model and polarimetric waves to obtain three components of the Stokes vector modeling, denoted as:

(6) $$s\;=\;m_{v}s_{v}+m_{s}s_{p}+ns_{n}$$

where $s_{n}$ is the randomly polarimetric Stokes vector and represents the noise term. $s_{v}$ and $s_{p}$ are the un-polarimetric and polarimetric Stokes vectors. $m_{v}$, $m_{s}$ and n are the corresponding total powers. It is shown that the noise term can be effectively removed by filtering techniques, and this decomposition uses a random dipole cloud instead of a body scattering model, then the decomposition model can be expressed as:

(7) $$s=m_{v}\left[\begin{array}{c} 1\\ \pm 0.5\\ 0\\ 0 \end{array}\right]+m_{s}\left[\begin{array}{c} 1\\ cos2\alpha \\ sin2\alpha cos\delta \\ sin2\alpha sin\delta \end{array}\right]$$

The four unknown parameters present in this model correspond to four observables in Stokes. The idea of solving this model lies in using the polarization information of the $s_{p}$ term. Where G is a commonly used matrix in Stokes vector in Equation (8):

(8) $$G=\left[\begin{array}{cccc} 1 & 0 & 0 & 0\\ 0 & -1 & 0 & 0\\ 0 & 0 & -1 & 0\\ 0 & 0 & 0 & -1 \end{array}\right]\to \left\{\begin{array}{c} det\left({C2}_{0}\right)\;=0\\ s_{p}^{T}Gs_{p}=0 \end{array}\right.$$

The volumetric power solution follows from Equation (9) quadratic form, evaluated using Equation (10) analytical expression:

(9) $$G{\left(s-m_{v}s_{v}\right)}^{T}G\left(s-m_{v}s_{v}\right)=0$$

(10) $$am_{v}^{2}+bm_{v}+c=0,\;\left\{\begin{array}{c} a={\;s}_{v}^{T}Gs_{v}=0.75\\ b=-2s^{T}Gs_{v}=-2s_{1\;}\pm \;0.5s_{2}\\ c=s^{T}Gs=s_{1}^{2}-s_{2}^{2}{\;-\;s}_{3}^{2}-s_{4}^{2} \end{array}\right.$$

In solving the quadratic equation, only one solution satisfies the law of conservation of energy $\left(m_{v}\leq s_{1}\right)$, so a unique $m_{v}$ can be found. In addition, this decomposition avoids the problem of solving for complex eigenvalues. The polarization component power $m_{s}$ can also be found from Equation (11).

(11) $$s\;-\;m_{v}s_{v}={\;m}_{s}s_{p}$$

In summary, based on the $C_{2}$ matrix extraction, we performed H/A/α/ decomposition and dual-polarization model-based decomposition for feature extraction. This method is founded on the eigenvalue analysis of the coherence matrix. This section extracted richer image features, which are beneficial for distinguishing different crops and can enhance classification accuracy.

### 2.6. Accuracy Assessment

In this experiment, classification performance was statistically evaluated via confusion matrix analysis [58], which is a routinely used tool in remote sensing image classification evaluation. The confusion matrix provides four key parameters:

Producer’s Accuracy (PA) [59]: The metric evaluates category-specific detection reliability by calculating the percentage of correctly predicted positive cases out of all genuine instances of that class.

User’s Accuracy (UA) [59]: This evaluation criterion characterizes precision as the relationship between correctly classified positive samples and the sum of all samples designated as positive by the classifier. It reflects the reliability of the model’s classification for a specific class.

Overall Accuracy (OA): The metric assesses overall model performance by measuring the percentage of all accurately identified cases across all classes in relation to the total observations, thereby measuring the model’s global classification performance.

Kappa Coefficient [60]: This metric provides an integrated assessment that incorporates both producer’s accuracy (recall) and user’s accuracy (precision) into a unified evaluation framework.

## 3. Results

### 3.1. Multi-Temporal Sentinel-2 Images Classification Results

**Figure 5:**
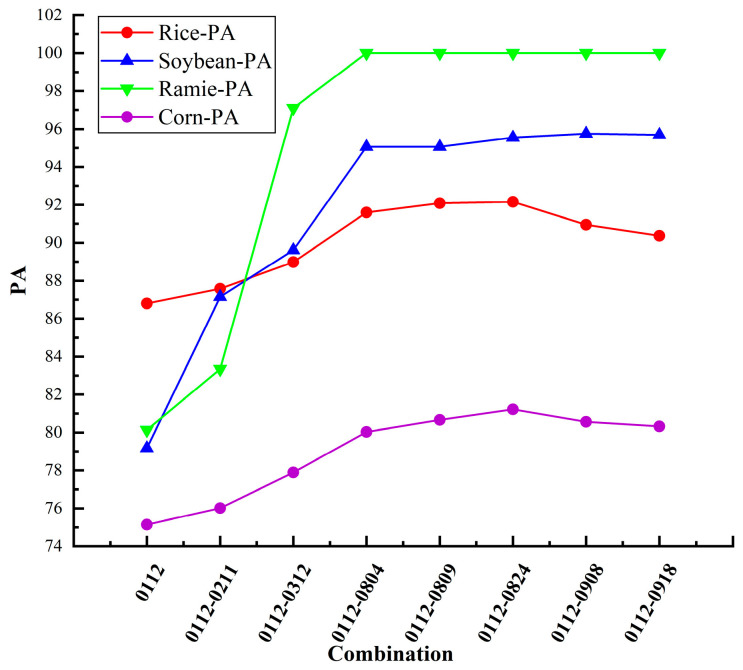
PA for the four crops versus the quantity of multi-temporal Sentinel-2 images.

To evaluate the temporal properties of Sentinel-2 optical imagery for crop classification within the study area, a series of experiments were designed and implemented. Using the multi-temporal Sentinel-2 dataset employed in this study—comprising 8 acquisitions from different phenological stages—we progressively combined images across time and quantitatively analyzed the resulting classification accuracy for each crop type. This accuracy was measured as a function of the number of images used, as illustrated in Figure 5. The PA for each crop is shown in Table 4. The results indicate that integrating four optical images markedly enhances the classification performance for all four crop types. Maximum accuracy is achieved with the inclusion of six images, beyond which further additions lead to a decline in performance.

To further investigate the contribution of early-season imagery (January and February), we conducted a comparative experiment by excluding these months from the input dataset. The results indicated a decrease in Overall Accuracy to 91.56% (Table 5). This finding confirms that despite the absence of standing crops, the spectral information from the fallow period—serving as a phenological baseline and aiding in the separation of cropland from evergreen vegetation—is essential for achieving optimal classification performance.

### 3.2. Multi-Temporal Sentinel-1 Intensity Images Classification Results

**Figure 6:**
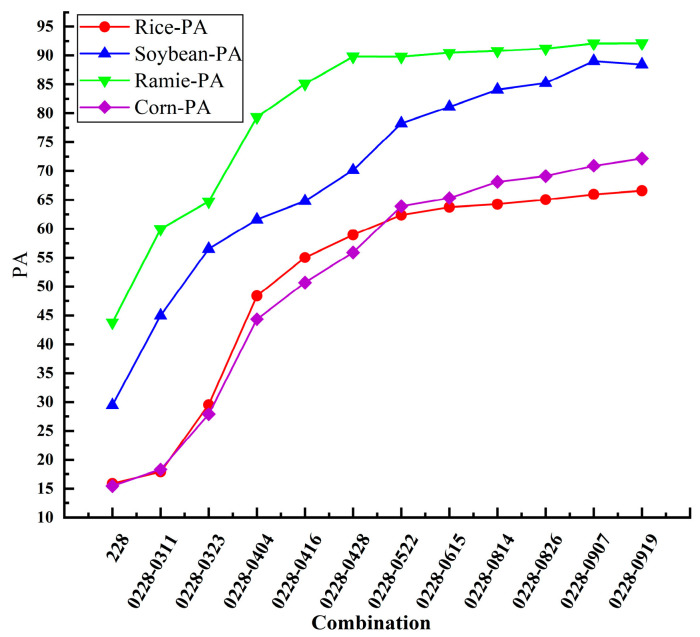
Variation in PA across four crop types with increasing number of Sentinel-1 intensity images.

To evaluate the contribution of Sentinel-1 dual-polarization decomposition features to crop classification, a controlled experiment was designed utilizing only preprocessed Sentinel-1 backscatter intensity images. The relevant results are shown in Figure 6, with the accuracy for each crop listed in Table 6. The subsequent classification results demonstrated that ramie and soybeans achieved recognition accuracies of 92.18% and 89.03%, respectively, when using a time series of 12 Sentinel-1 intensity images. However, the accuracy of rice, the main crop in the planting area, was only 66.59%. Therefore, classification based solely on Sentinel-1 intensity images cannot meet application requirements. It is necessary to extract more features based on polarimetric SAR data to enhance the classification effect.

When only the 12 preprocessed intensity images from Sentinel-1 were used, the classification outcome proved inadequate. The OA of the classification results is 65.00%, the Kappa coefficient is 0.52. The PA values of the four crops including rice, soybean, corn, and ramie are 66.59%, 89.03%, 72.18%, 92.18%, respectively. Only utilizing the backscattering products of Sentinel-1 does not achieve good classification results. Consequently, in subsequent experiments, we leveraged dual-polarization decomposition to enhance the feature space of Sentinel-1 imagery and more comprehensively evaluated its efficacy in crop classification.

### 3.3. Multi-Temporal Sentinel-1 Features Images Classification Results

**Figure 7:**
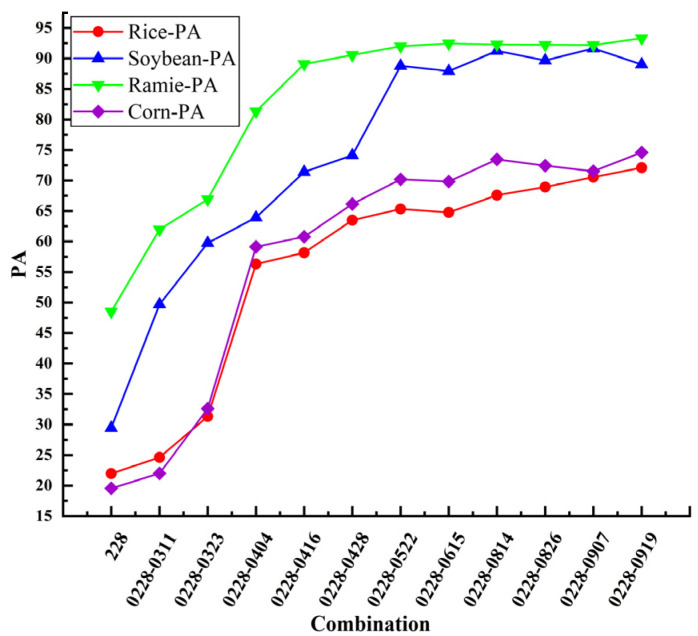
Variation in PA across four crop types with increasing number of Sentinel-1 features images.

**Figure 8:**
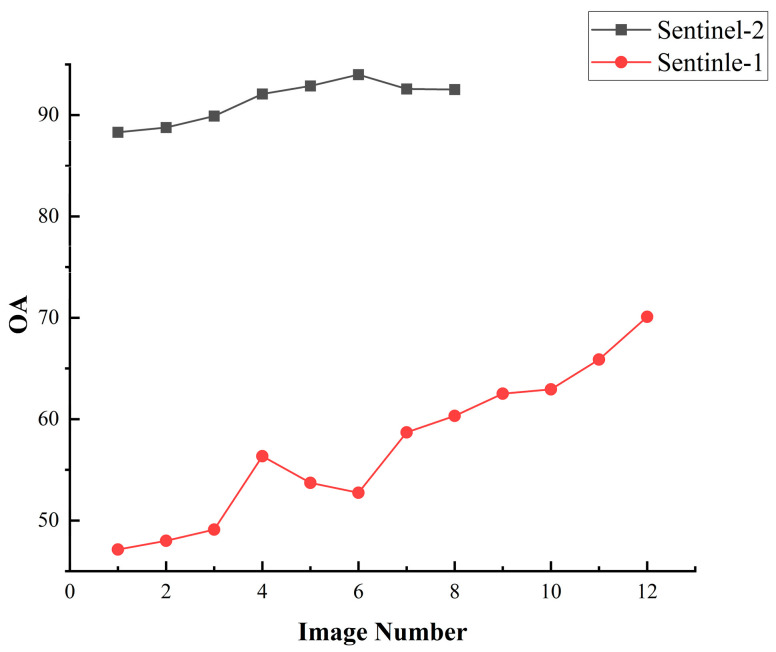
Variation in OA between multi-temporal optical data and SAR data.

To improve the precision of crop type identification with Sentinel-1 imagery, based on the $C_{2}$ matrix, we extracted the polarization decomposition features of Sentinel-1. And the temporal features of crop classification of Sentinel-1 data were obtained by using 12 Sentinel-1 images for crop classification. The classification accuracy for each crop is shown in Table 7. We incrementally aggregated multi-temporal images and quantitatively assessed the per-crop classification accuracy, evaluated as functions of image count variations, as shown in Figure 7. The overall classification accuracy variation for multi-temporal optical data and multi-temporal SAR data is shown in Figure 8.

Based on experimental results, it demonstrated that after adding the polarization decomposition feature, the classification accuracies of the four crops are improved to different degrees compared with the classification results of Sentinel-1 intensity images. Among them, the classification accuracy of rice is improved by 5.50%, soybean by 2.60%, corn by 2.41%, and ramie by1.35%. Notably, rice displayed the most pronounced enhancement, which is particularly valuable for operational monitoring since it is the most extensively cultivated crop in the region.

### 3.4. The Best Crop Classification Strategy

With the time-series classification curves from both satellite systems now established, we proceeded to conduct comparative experiments aimed at developing an optimal crop classification strategy for the region.

**Figure 9:**
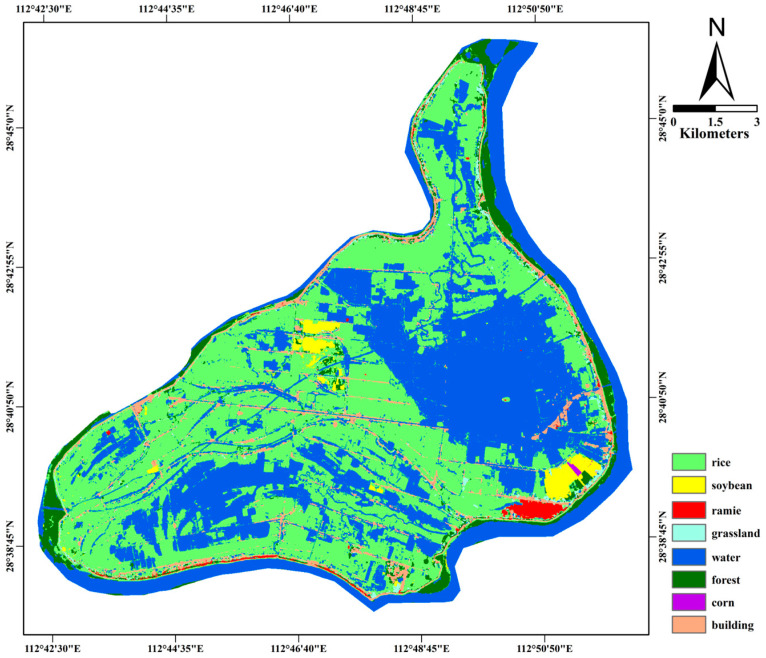
The result for the best classification combination.

**Figure 10:**
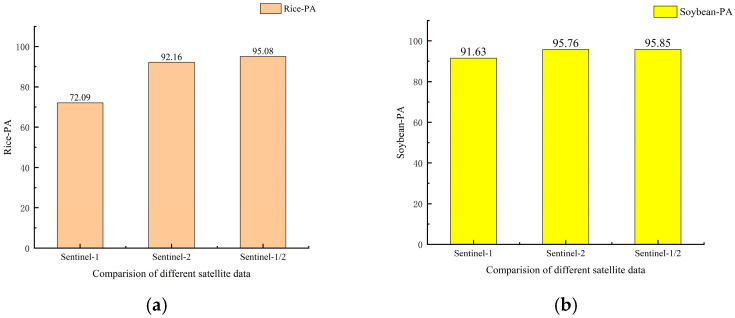

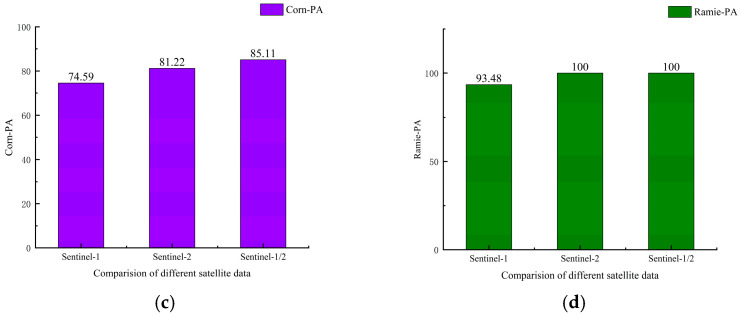
Comparison of different satellite data ((**a**–**d**) represent the classification accuracy of rice, soybeans, corn, and ramie, respectively, under different datasets.).

Section 3.1 and Section 3.3 have illustrated the OA of classifications derived from Sentinel-1 and Sentinel-2 imagery. The results demonstrate that the classification accuracy of optical images initially rises with the inclusion of more temporal data, peaks at an optimal number of images, and subsequently declines with further additions. The overall classification accuracy of optical images peaks at 6 images. However, the overall accuracy of SAR data increases with the number of images and reaches a peak at 12 images. Several studies have demonstrated that combining Sentinel-1/2 time series could significantly improves crop discrimination accuracy [61,62,63]. Therefore, all Sentinel-1 feature images and the combination of Sentinel-2 images from 12 January 2024 to 24 August 2024 were selected for crop classification as the most applicable classification scheme for the experimental area. A thematic map illustrating the output of the best classification strategy is provided in Figure 9 and Confusion Matrix as shown in Table 8. The performance of each crop under the optimal classification strategy is shown in Figure 10.

A comparison of the accuracy matrices reveals that the overall classification accuracy with polarimetric decomposition features reaches 94.20%, representing only a 0.20% improvement over the best result achieved using optical data alone. Although this increase is modest, focusing on specific crop types reveals more significant improvements. Notably, the classification accuracy for rice improved by 2.92%, and for corn, it increased by 3.89%. This enhancement, while not substantial in terms of overall accuracy, is particularly significant in practical applications, especially under challenging conditions where optical imagery is either absent or of poor quality. The study area’s climatic conditions, characterized by persistent precipitation and prolonged cloud cover during the rice and corn growing seasons, as well as intercropping practices, make it difficult to achieve reliable classification when relying solely on optical imagery. The integration of SAR data, particularly the polarimetric decomposition features, effectively addresses these challenges and improves the classification accuracy of specific crops in the study area, demonstrating its crucial application value when optical data quality is compromised or missing.

## 4. Discussion

### 4.1. Main Findings and Explanations

The rice and corn growing season in the study area is affected by rainy weather, making it difficult to achieve the objective of the task of extracting rice and corn growing areas by relying only on optical remote sensing data. Therefore, this case study systematically quantifies dual-polarization SAR’s efficacy for crop phenology monitoring under cloud-constrained environments and decomposition features in the extraction of crop planting areas, demonstrating that integrating multi-temporal Sentinel-2 optical data with Sentinel-1 polarimetric decomposition features substantially improves crop classification accuracy in crops landscapes. Three principal findings include:

Non-linear temporal effects: Classification accuracy showed significant improvement when using four multi-temporal images acquired between 12 January and 9 August 2024. This improvement likely stems from the close temporal alignment of the August 9 image with the field data collection period, ensuring high feature similarity between ground samples and satellite observations.

**Figure 11:**
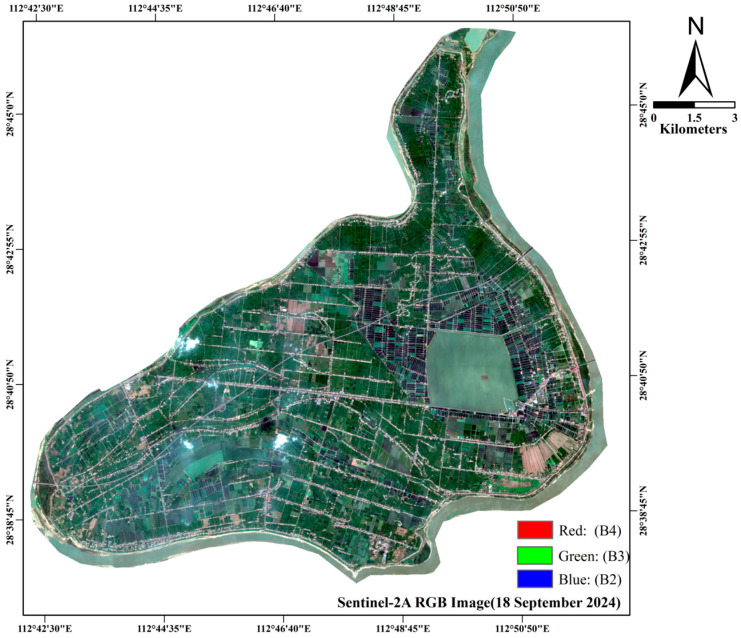
Cloud cover on 18 September 2024 is present on the image.

However, as additional images were incorporated, the overall accuracy plateaued and slightly decreased upon including the 18 September 2024 image. Analysis of this image (Figure 11) revealed cloud cover obstructing ground features in the study area. Thus, accuracy improvements depend more critically on image quality (e.g., radiometric consistency and noise levels) than merely on increasing the number of images.

**Figure 12:**
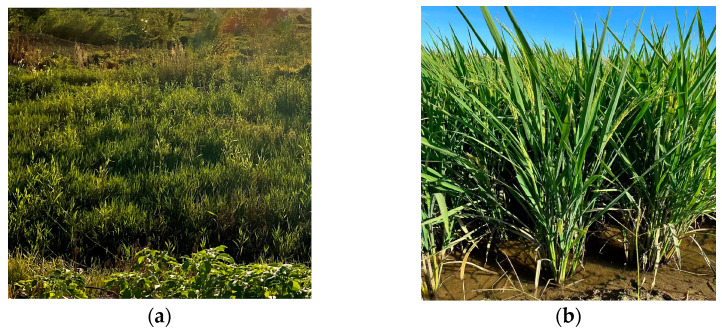
Field photos in the study area. (**a**) Grass; (**b**) Rice.

The spectral signatures of rice during the mid-growth stage are highly similar to those of dense grasses, as shown in Figure 12, leading to potential misclassification in optical imagery. This spectral ambiguity is further quantified through sample separability analysis. Jeffries–Matusita Distance [64,65], a standardized remote sensing metric for class distinguishability (Equation (12)), ranges from 0 (inseparable) to 2 (fully separable). According to Richards and Jia [65], a JM distance greater than 1.90 is typically required to indicate good separability. Consequently, the obtained results suggest that the spectral separability between rice and water, as well as between rice and grass, is insufficient, as shown in Table 9. However, in the practice of remote sensing crop classification, a JM distance value exceeding 1.9 is typically required to indicate distinct separability between two classes.

(12) $$J_{M\left(p,q\right)}=\sqrt{2\left(1\;{-\;e}^{-D_{B\left(p,q\right)}}\right)}$$

where p and q, respectively, denote the two probability distribution created by a feature.

**Figure 13:**
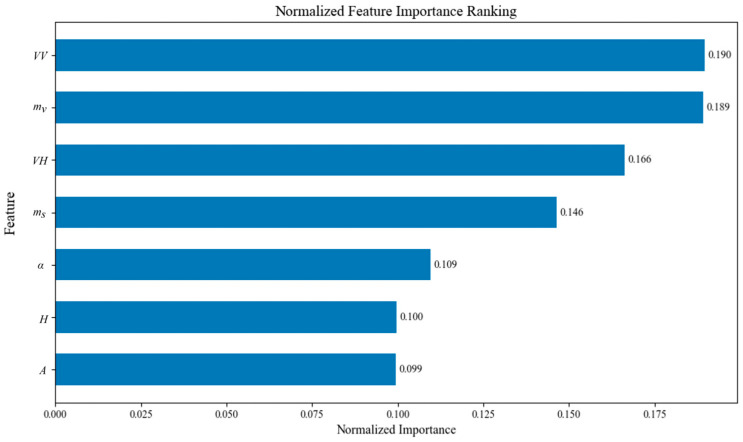
Normalized feature importance ranking results based on seven SAR features.

Due to persistent cloud and rainfall interference during the growing season in the study area (as depicted in Figure 11), merely increasing the number of stacked optical images does not significantly enhance crop classification accuracy. Furthermore, it remains challenging to acquire optical imagery that concurrently satisfies the requirements of both minimal cloud obstruction and temporal continuity. Considering the unique geographical location of the study area and its high susceptibility to frequent cloud cover and rainfall, this case study focuses on investigating the importance of polarimetric SAR features in supplementing optical data to optimize crop classification performance. Therefore, only the feature importance of SAR-derived parameters was analyzed, as shown in Figure 13. The polarimetric decomposition features selected in this study—entropy (H), anisotropy (A), and mean scattering angle (α) from the Cloude–Pottier decomposition, together with the model-based dual-polarization parameters of surface scattering power ($m_{s}$) and volume scattering power ($m_{v}$)—have been demonstrated to provide significant contributions to crop classification. Specifically, H, A, and α describe the randomness, relative importance of scattering mechanisms, and the dominant scattering type of targets, respectively. Previous studies have shown that these parameters are highly sensitive to vegetation structure and phenological stages [66,67], enabling the effective discrimination of different crop types beyond the capability of simple backscatter coefficients. On the other hand, the dual-polarization model-based decomposition parameters $m_{s}$ and $m_{v}$ provide physical insights into surface and volume scattering contributions [57,68]. $m_{s}$ is closely associated with soil–plant interface reflections, whereas $m_{v}$ reflects canopy volume scattering, which is strongly related to crop biomass and height. Recent studies have confirmed that these parameters are effective indicators of crop phenology and structural variability, thereby improving class separability when integrated into classification models.

Therefore, the combination of H, A, α, $m_{s}$, and $m_{v}$ offers a more comprehensive description of crop scattering mechanisms, enabling higher classification accuracy compared to the use of intensity-only features.

### 4.2. Comparison with Previous Work

Our classification strategy shows good results (OA = 94.20%, Kappa = 0.91) and is comparable with previous studies. For instance, Veloso et al. [11] reported 89% OA for European crops using Sentinel-1/2 data but without incorporating polarimetric features. Zhao et al. [27] achieved 91% OA using Sentinel-1 coherence features, though their approach required 15 acquisitions. In contrast, our method attained higher accuracy with fewer images (12 SAR acquisitions) by strategically leveraging physical scattering mechanisms ($m_{s}$, $m_{v}$) and phenological trajectories. Bargiel et al. [69] developed an innovative classification approach that integrates time-series radar data and accounts for climate-induced variations in agricultural areas; however, most of his studies focused on non-cereal crops [70,71]. Wang et al. [72] demonstrated the potential of GF-3 full-polarimetric data for dryland crop classification. Nevertheless, compared to full-polarimetric data, Sentinel data offers free accessibility and wider availability, making it more practical for large-scale applications. Furthermore, polarimetric decomposition features provide unique advantages in identifying surface scattering mechanisms of crops, significantly enhancing the accuracy of rice area extraction in our study region.

### 4.3. Limitations and Future Work

Although the overall accuracy reached 94.20%, there remain areas for improvement and several limitations. First, regarding timeliness and sample abundance: the field data were not temporally characterized and were concentrated only at the time of collection (July 2024). Future studies should aim to collect time-matched ground samples across the growing season to better capture crop dynamics. Second, the limited quantity of training samples for certain crops, such as corn, poses a constraint. in machine learning. Class imbalance is a common challenge, and typical solutions include data-level techniques such as over-sampling (e.g., Synthetic Minority Over-sampling Technique (SMOTE)) and under-sampling, as well as algorithmic approaches like weighted loss functions (e.g., Focal Loss) and cost-sensitive learning [73,74,75]. However, it is important to note that the samples used in this study were directly collected from the field in the experimental area, ensuring the authenticity and real-world relevance of the dataset. To maintain the integrity of the experiment and reflect actual agricultural conditions, we used the original samples without augmentation for classification. This approach prioritizes the accuracy of results based on real, ground-truth data, rather than relying on synthetic or extrapolated samples. It is worth noting that the random split of pixels may introduce spatial autocorrelation effects compared to object-based independent validation, potentially resulting in slightly higher accuracy estimates. Third, the incorporation of deep learning approaches may further optimize the classification framework in future studies. Advanced architectures, such as LSTM, GRU, and semantic segmentation networks, have been successfully applied to time-series SAR and optical data for refined crop mapping [76,77,78]. Models such as Convolutional Neural Networks (CNNs), Long Short-Term Memory networks (LSTMs), and Transformer-based architectures have demonstrated strong capabilities in exploiting the spatial, temporal, and polarimetric characteristics of Sentinel data. For example, Liu et al. [67] applied a patch-based neural network using polarization decomposition features from Sentinel-1 time series and achieved significantly higher classification accuracy than intensity-based methods. Similarly, Qi et al. [79] reported that Transformer models outperformed CNNs and LSTMs when integrating Sentinel-1 and Sentinel-2 imagery for multi-crop mapping. More recently, Gallo et al. [80] demonstrated that Transformer architectures such as Swin UNETR improved temporal segmentation stability in satellite time-series classification, while Patnala et al. [81] proposed a bi-modal self-supervised framework to alleviate the dependence on large labeled datasets. By integrating the dual-polarization decomposition features analyzed in this study, deep learning methods could potentially capture more comprehensive hierarchical scattering representations, achieving enhanced crop discrimination and better generalization across regions. Last, although the inclusion of Sentinel-1 SAR data provides all-weather observation capabilities and compensates for data gaps, the rigorous cloud screening of Sentinel-2 imagery inevitably disrupted the continuity of the time series. In this study, the absence of high-quality optical images during key phenological stages (e.g., the specific heading or ripening stages of paddy rice) may have led to the loss of critical spectral signatures that are vital for distinguishing spectrally similar crops. While the polarimetric decomposition features of SAR data offer supplementary structural information, they cannot fully replace the rich spectral information provided by optical bands, especially for crops with similar geometric structures but distinct pigment contents.

## 5. Conclusions

In this study, the crop classification capabilities of multi-temporal Sentinel-1 and Sentinel-2 data were independently evaluated. Particular emphasis was placed on assessing the contribution of dual-polarization model-based decomposition features for crop classification. An integrated classification framework tailored to the study area was developed using Sentinel imagery. By analyzing the effect of multi-temporal data stacking on classification outcomes, the following conclusions were drawn:

(1) Stacking multi-temporal SAR or optical images generally improves overall classification accuracy; however, the improvement is not strictly linear and may even decline after reaching saturation.
(2) Optical imagery can adequately support crop classification when free from clouds or other masking effects. In cloudy conditions, classification accuracy declines, and incorporating SAR decomposition features can effectively enhance performance.
(3) Parameters derived from dual-polarization model–based decomposition efficiently improved classification accuracy, particularly for identifying rice and soybean crops.

## Figures and Tables

**Table 1:** Satellite information for Sentinel-1.

Parameter	Information
Satellite name	Sentinel-1A
Incidence range	30.3–42.8°
Azimuth Pixel Spacing	13.98 m
Sensor type	Synthetic Aperture Radar (SAR)
Operating frequency	C (5.4 GHz)
Polarimetric mode	VV, VH.
Imaging mode	Interferometric Wide Swath (IW)
Available data	20240228, 20240311, 20240323, 20240404, 20240416, 20240428, 20240522, 20240615, 20240814, 20240826, 20240907, 20240919
Data format	Single Look Complex (SLC)

**Table 2:** Satellite information for Sentinel-2.

Sentinel-2 Bands Information	Centre Wavelength (μm)	Resolution (m)
B1	0.44	60
B2	0.49	10
B3	0.56	10
B4	0.67	10
B5	0.71	20
B6	0.74	20
B7	0.78	20
B8	0.84	10
B8A	0.87	20
B9	0.95	60
B10	1.38	60
B11	1.61	20
B12	2.19	20
Available data	20240112, 20240211, 20240312, 20240804, 20240809, 20240824, 20240908, 20240918.	
Level	L2A	

**Table 3:** Number of samples in each category of field collection data.

Category	Training Polygons	Training Pixels	Testing Polygons	Testing Pixels
forest	40	5230	2	2292
water	506	35,374	58	13,928
soybean	197	3634	54	1416
ramie	55	1917	12	793
rice	1028	28,215	199	11,270
grassland	99	1537	41	679
building	88	942	74	582
corn	14	403	5	181

**Table 4:** Matrix of variation in classification accuracy with number of Sentinel-2 images for four crops.

Combination	Number of Image	Rice-PA	Soybean-PA	Ramie-PA	Corn-PA
0112	1	86.8	79.17	80.13	75.14
0112-0211	2	87.58	87.15	83.35	76.01
0112-0312	3	88.98	89.62	97.1	77.89
0112-0804	4	91.6	95.06	100	80.03
0112-0809	5	92.09	95.06	100	80.66
0112-0824	6	92.16	95.55	100	81.22
0112-0908	7	90.95	95.76	100	80.56
0112-0918	8	90.37	95.69	100	80.32

**Table 5:** Classification accuracy for the four crops and overall accuracy after removing optical images from January to March.

Combination	Number of Image	Rice-PA	Soybean-PA	Ramie-PA	Corn-PA
0312-0918	6	87.52	96.75	99.75	80.11
OA	91.56				
Kappa	0.87				

**Table 6:** Variation in PA across four crop types with increasing number of Sentinel-1 intensity images.

Combination	Number of Image	Rice-PA	Soybean-PA	Ramie-PA	Corn-PA
0228	1	15.87	29.45	43.77	15.44
0228-0311	2	17.91	44.98	59.96	18.33
0228-0323	3	29.54	56.52	64.72	27.88
0228-0404	4	48.37	61.59	79.32	44.34
0228-0416	5	55.02	64.8	85.1	50.67
0228-0428	6	58.94	70.14	89.79	55.9
0228-0522	7	62.36	78.23	89.78	63.88
0228-0615	8	63.73	81.12	90.46	65.32
0228-0814	9	64.26	84.09	90.78	68.12
0228-0826	10	65.03	85.22	91.22	69.09
0228-0907	11	65.95	89.03	92.18	70.91
0228-0919	12	66.59	88.43	91.8	72.18

**Table 7:** Matrix of variation in classification accuracy with number of Sentinel-1 features images for four crops.

Combination	Number of Image	Rice-PA	Soybean-PA	Ramie-PA	Corn-PA
0228	1	21.96	29.45	48.5	19.55
0228-0311	2	24.62	49.68	61.96	21.99
0228-0323	3	31.31	59.75	66.92	32.6
0228-0404	4	56.33	63.93	81.32	59.12
0228-0416	5	58.17	71.41	89.1	60.77
0228-0428	6	63.49	74.14	90.56	66.15
0228-0522	7	65.34	88.8	92	70.18
0228-0615	8	64.76	87.92	92.46	69.84
0228-0814	9	67.6	91.24	92.29	73.45
0228-0826	10	68.93	89.67	92.23	72.43
0228-0907	11	70.55	91.63	92.19	71.51
0228-0919	12	72.09	89.03	93.48	74.59

**Table 8:** Confusion matrix of the best classification result. (1, rice; 2, soybean; 3, ramie; 4, grassland; 5, water; 6, forest; 7, corn; 8, building).

Ground Truth										
Class	1	2	3	4	5	6	7	8	PA	UA
1	10,716	22	0	221	332	0	3	38	95.08	94.56
2	132	1357	0	62	0	0	33	0	95.85	85.67
3	0	0	793	3	23	0	0	0	100.00	96.83
4	89	13	0	283	6	0	0	1	41.68	72.19
5	251	0	0	33	13,566	351	0	13	97.40	95.44
6	63	2	0	71	1	1941	0	0	84.69	93.41
7	17	0	0	0	0	0	145	0	85.11	89.51
8	2	22	0	6	0	0	0	530	91.07	94.64
OA	94.20									
Kappa	0.91									

**Table 9:** Comparison of separability between classes.

Class	Rice	Water	Grassland
rice		1.31	0.97
water	1.31		
grassland		1.92	

## Data Availability

The original contributions presented in this study are included in the article. Further inquiries can be directed to the corresponding author.
